# *Mycobacterium avium* infection induced PD-L1 overexpression in macrophages: a potential involvement with immune escape

**DOI:** 10.1038/s41419-025-08165-z

**Published:** 2026-01-09

**Authors:** Hiromu Yano, Yukio Fujiwara, Remi Mito, Cheng Pan, Katsuhiko Ono, Kosuke Imamura, Takuro Niidome, Tomohiro Sawa, Masahiro Yamamoto, Takuro Sakagami, Yoshihiro Komohara

**Affiliations:** 1 Department of Tumor Pathology, Graduate School of Health Sciences, Kumamoto University, 4-24-1 Kuhonji, Chuo-ku, Kumamoto 862-0976 Japan; 2 Department of Cell Pathology, Graduate School of Medical Sciences, Kumamoto University, 1-1-1 Honjo, Chuo-ku, Kumamoto 860-8556 Japan; 3 Department of Respiratory Medicine, Graduate School of Medical Sciences, Kumamoto University, 1-1-1 Honjo, Chuo-ku, Kumamoto 860-8556 Japan; 4 Department of Microbiology, Graduate School of Medical Sciences, Kumamoto University, 1-1-1 Honjo, Chuo-ku, Kumamoto 860-8556 Japan; 5 Faculty of Advanced Science and Technology, Kumamoto University, 2-39-1 Kurokami, Chuo-ku, Kumamoto 860-8555 Japan; 6 Center for Biomedical Laboratory Science, Faculty of Life Sciences, Kumamoto University, 4-24-1 Kuhonji, Chuo-ku, Kumamoto 862-0976 Japan; 7 Center for Metabolic Regulation of Healthy Aging, Kumamoto University, 1-1-1 Honjo, Chuo-ku, Kumamoto 860-8556 Japan

**Keywords:** Immune evasion, Bacterial infection

## Abstract

Non-tuberculous mycobacteria (NTM) infections are difficult to cure completely with current treatments, and no specific drugs are available. However, recent reports have indicated that immune checkpoint inhibitors may effectively treat pulmonary NTM infections. In this study, we investigated the expression of immune checkpoint molecules in macrophages, the host cells of NTM, and assessed their impact on the microenvironment of infected lesions. Bulk-RNA sequencing and western blot analyses revealed that macrophages infected with *Mycobacterium avium*, an NTM species, exhibited a pro-inflammatory phenotype and increased PD-L1 expression. Additionally, immunostaining of an NTM-infected mouse model and human tissues showed that increased PD-L1 expression in macrophages was associated with decreased T cell infiltration and increased T cell exhaustion (upregulated PD-1 expression) within infected lesions. These findings suggest that NTM infections evade cellular immunity by enhancing PD-L1 expression in macrophages. Therefore, PD-L1 inhibition may be a promising therapeutic strategy against NTM infections.

## Introduction

Non-tuberculous mycobacteria (NTM) are opportunistic bacteria found abundantly in soil and water, including both natural and plumbing-related water sources [1]. The incidence of NTM infections is rising globally [2], with Japan experiencing particularly higher rates than other countries [3, 4]. Additionally, an increase in mortality rates associated with NTM infections has been reported [3]. In Japan, *Mycobacterium avium* complex (MAC), which includes *Mycobacterium avium* (*M. avium*) and *Mycobacterium intracellulare*, is the most frequent cause of pulmonary NTM. Typical symptoms include persistent cough, phlegm, and occasionally blood-streaked sputum. Pharmacotherapy for pulmonary MAC typically involves a multidrug regimen comprising rifampicin, ethambutol, and clarithromycin (CAM). However, this regimen is often less effective for treating bronchial and pulmonary lesions. Consequently, the treatment may require a year or more to achieve a cure, and drug resistance is frequently observed. Furthermore, the prognosis of CAM-resistant cases is comparable with that of multidrug-resistant tuberculosis (TB) [5], highlighting the importance of managing the disease to minimize resistance development. Although NTMs are generally not transmitted from human to human, a recent study has suggested that *Mycobacterium abscessus* (*M. abscessus*), one of the NTMs, may be transmitted among patients with cystic fibrosis, which has drawn significant attention [6]. The British Thoracic Society guidelines for the management of NTM recommend implementing infection control measures against *M. abscessus* in both inpatient and outpatient settings among patients with cystic fibrosis [7]. Given these factors, the prevalence of NTM diseases is anticipated to continue to increase. Therefore, it is crucial to understand the pathophysiology of NTM, develop simple and sensitive diagnostic methods, and establish optimal treatment strategies.

The infection by intracellular parasitic NTMs involves an immune response, with macrophage-mediated killing mechanisms playing a crucial role. Enhancing the bactericidal activity of macrophages against mycobacteria requires interactions among immune cells centered around the infected macrophages, particularly through activating cellular immune mechanisms via cytokine networks [8]. However, NTMs possess extremely lipid-rich and thick cell walls that render them highly resistant to bactericidal mechanisms. This resistance allows NTMs to evade macrophage-mediated cell death and proliferate within these cells [9]. Furthermore, recent reports indicate that the expression of programmed cell death-1 (PD-1) and its ligand, programmed cell death-1 ligand 1 (PD-L1), in the lymphocytes of patients with pulmonary NTM disease is elevated compared with that in healthy individuals [10]. PD-L1 is expressed in macrophages and vascular endothelial cells, and its expression is upregulated by inflammation [11]. PD-L1 binding to PD-1 suppresses T cell activation and regulates the immune response [12]. PD-L1 is expressed on various tumor cells and promotes immune escape by suppressing T cell activation. Owing to this, PD-1/PD-L1 antibodies have shown promising therapeutic effects in cancer immunotherapy. Moreover, studies have reported the resolution of NTM infections during cancer immunotherapy [13], suggesting that PD-1/PD-L1-mediated immunosuppression may contribute to NTM infection in macrophages. In this study, we investigated the immune escape mechanisms of NTMs via PD-L1 expression in macrophages, the host cells of NTMs.

## Material and methods

### Human tissue samples

Human skin tissues infected with NTM and peritoneal tissues infected with TB, used in this study were collected at Izumi General Medical Center. Tissue samples were fixed in 10% neutral-buffered formalin and embedded in paraffin.

### Chemicals

The STAT1 inhibitor fludarabine was purchased from Selleck Chemicals (Houston, TX, USA). The NF-κB inhibitor Bay 11-7082 was purchased from FUJIFILM Wako Pure Chemical Corporation (Osaka, Japan). The p38 inhibitor adezmapimod (SB203580) was purchased from Selleck Chemicals. The JNK inhibitor SP600125 was purchased from LC Laboratories (Waltham, MA, USA). IFN-γ was purchased from FUJIFILM Wako Pure Chemical Corporation. Anti-mouse PD-L1 (B7-H1)-InVivo (clone: 10F.9G2) and rat IgG2b isotype control-InVivo (clone: LTF-2) were purchased from Selleck Chemicals.

### Macrophage culture

Peripheral blood samples were collected from healthy volunteers after obtaining written informed consent for sample collection and subsequent experimental use. Peripheral blood mononuclear cells (PBMCs) were isolated using Lymphoprep™ (STEMCELL Technologies Inc., Vancouver, Canada). Isolated monocytes were cultured in Corning Primaria tissue culture dishes (Corning Inc., Corning, NY, USA) with 10 ng/mL recombinant human M-CSF (FUJIFILM Wako Pure Chemical Corporation) for 7 days to induce differentiation into human monocyte-derived macrophages (HMDMs). These HMDMs were maintained in DMEM (low glucose) (FUJIFILM Wako Pure Chemical Corporation) supplemented with 2% fetal bovine serum (FBS) and 100 µg/mL penicillin–streptomycin solution.

### Bacterial growth and cell stimulation

*M. avium* and *M. avium* harboring pGFPHYG2 (#30173, Addgene, Watertown, MA, USA) (GFP-expressing *M. avium*) were cultured in MycoBroth (Kyokuto Pharmaceutical, Tokyo, Japan) at 37 °C with 5% CO_2_. When the bacteria reached the exponential growth phase, the optical density of the bacterial solution was measured using a spectrophotometer (ABS-B470; Kyoritsu Chemical Check Lab, Tokyo, Japan). For cell stimulation, HMDMs were treated with *M. avium* at a multiplicity of infection (MOI) of 20 in DMEM (low glucose) (FUJIFILM Wako Pure Chemical Corporation) supplemented with 2% FBS for 24 h. After stimulation, *M. avium* was removed from the culture supernatant, and the HMDMs were cultured in a regular medium for 6 days.

### Electron microscopy

HMDMs were cultured in the presence of *M. avium*. After incubation, the HMDMs were fixed in 2.5% glutaraldehyde in 0.1 M cacodylate buffer for 1 h and then post-fixed in 1% osmium tetroxide. After dehydration in a graded series of ethanol and propylene oxide solutions, the samples were embedded in Epon 812. Ultrathin sections were cut using an ultramicrotome (EM UC7; Leica, Wetzlar, Germany), stained with uranyl acetate and lead citrate, and observed using a transmission electron microscope (TEM) (H-7700; Hitachi, Tokyo, Japan).

### Bulk RNA-sequencing data analysis

After HMDMs were cultured with *M. avium* for 24 h, *M. avium* was removed from the culture supernatant, and the HMDMs were subsequently cultured in a regular medium for 6 days. Following stimulation, HMDMs were immersed in a cell preservation solution and transported to the contracted company. Bulk RNA-sequencing was outsourced to CyberOmix (Kyoto, Japan) according to the protocol provided by the manufacturer.

The R and edgeR packages were used to analyze bulk RNA-seq data. The read count table was loaded with the DGEList function as the DGE list object. Normalization factors accounting for the library size of each sample were calculated using the TMM method with the calcNormFactors function. A general linear model (GLM) was used for differentially expressed gene analysis (DEG analysis) between control and infected macrophage samples. The common dispersion was estimated using the estimateDisp function. The glmQLFit function was used to fit a negative binomial GLM, and whether the genes were DEGs was tested using the glmQLFTest function.

For the heatmap, the CPM values normalized using the TMM method for each gene were extracted using the function CPM. The values were logarithmically transformed and scaled to z-scores across all samples. The results of the DEG analysis were used for gene set enrichment analysis (GSEA) using the packages org.Hs.eg.db and ClusterProfiler. The average log2 (fold change) × log (FDR) values were used to rank genes. The gseGO function was used to conduct GSEA for the gene sets of the gene ontology biological process.

### Real-time quantitative PCR

Total RNA was isolated using the RNAiso Plus reagent and reverse-transcribed using the PrimeScript RT reagent kit (Takara Bio Inc., Shiga, Japan). Real-time quantitative PCR was conducted using TaqMan polymerase with SYBR green fluorescence (Takara Bio Inc.) and an ABI PRISM 7300 sequence detector (Applied Biosystems (Thermo Fisher Scientific), Waltham, MA, USA). The following primers were used: PD-L1, sense 5′-AAA.TGG.AAC.CTG.GCG.AAA.GC-3′, antisense 5′-GAT.TCA.GAG.CTA.CCC.CTC.AGG.CAT.TT-3′; β-actin, sense 5′-ATT.CCT.ATG.TGG.GCG.ACG.AG-3′, antisense 5′-AAG.GTG.TGG.TGC.CAG.ATT.TTC-3′.

### Western blot analysis

HMDMs were lysed in ice-cold lysis buffer supplemented with a protease inhibitor cocktail (Sigma-Aldrich, St. Louis, MO, USA). Proteins were separated by SDS–PAGE and transferred onto polyvinylidene fluoride membranes, which were incubated with primary antibodies including anti-PD-L1 (#13684, Cell Signaling Technology [CST], Danvers, MA, USA), anti-p38 MAPK (#8690, CST), anti-phospho-p38 MAPK (#4511, CST), anti-JNK1/2 (#9252, CST), anti-phospho-JNK1/2 (#9251, CST), anti-STAT1 (#9175, CST), anti-phospho-STAT1 (#9167, CST), anti-NF-κB (#8242, CST), anti-phospho-NF-κB (#3033, CST), and anti-β-actin (sc-47778, Santa Cruz Biotechnology, Dallas, TX, USA). HRP-conjugated secondary antibodies included goat anti-mouse IgG (#7076, CST) and goat anti-rabbit IgG (#7074, CST). Immunoreactive bands were visualized using a Pierce western blotting Substrate Plus Kit (Thermo Fisher Scientific, Waltham, MA, USA) and Amersham Imager 680 (Fujifilm, Tokyo, Japan).

### Flow cytometry

Single-cell suspensions were prepared from mouse lung tissue and treated with FcR-blocking reagent (BioLegend Inc., San Diego, CA, USA). The cells were stained with PerCP-Cy5.5-labeled anti-CD69 antibodies (clone H1.2F3, BioLegend), BV-421-labeled anti-PD-1 antibodies (clone 29F.1A12, BioLegend), and isotype-matched control antibodies (BioLegend). Stained cells were analyzed using a FACSverse flow cytometer (Becton Dickinson and Company, Franklin Lake, NJ, USA) and the FACSuite software program (Becton Dickinson).

### Immunohistochemistry

Human specimens were cut into 3-µm sections. The sections were deparaffinized and rehydrated, followed by antigen retrieval, endogenous peroxidase removal, and blocking with protein block. The primary antibodies used were anti-PD-L1 (#SK006, Agilent Technologies, Santa Clara, CA, USA), anti-PD-1 (#43248, CST), anti-CD4 (#413181, Nichirei Biosciences, Tokyo, Japan), anti-CD8 (#413201, Nichirei Biosciences), and anti-Iba1 (#019-19741, FUJIFILM Wako Pure Chemical Corporation). Samples were then incubated with peroxidase-labeled goat anti-mouse or rabbit secondary antibodies (anti-mouse, #424132; anti-rabbit, #424142; Nichirei Biosciences). Immunoreactions were visualized using the Diaminobenzidine Substrate Kit (#425011; Nichirei Biosciences).

Mouse specimens were similarly processed into 3-µm sections. The sections were subjected to deparaffinization, rehydration, followed by antigen retrieval, endogenous peroxidase removal, and blocking with protein block. The primary antibodies used were anti-PD-L1 (#64988, CST), anti-PD-1 (ab214421, Abcam, Cambridge, UK), anti-CD8 (#98941, CST), anti-CD25 (#36128, CST), anti-Ki-67 (#418071, Nichirei Biosciences) and anti-Iba1 (#019-19741, FUJIFILM Wako Pure Chemical Corporation). Samples were then incubated with peroxidase-labeled goat anti-rabbit secondary antibodies (#413341; Nichirei Biosciences). Immunoreactions were visualized using the Diaminobenzidine Substrate Kit (#425011; Nichirei Biosciences).

### Immunofluorescence staining

HMDMs were cultured in 6-well plates with *M. avium*. After infection, HMDMs were fixed in 4% paraformaldehyde for 15 min at room temperature. The cells were then incubated with primary antibodies against Iba1 (#019-19741, FUJIFILM Wako Pure Chemical Corporation) and PD-L1 (ab228462, Abcam) for 2 h at room temperature. Following incubation with the primary antibody, the cells were treated with the corresponding Alexa Fluor 594-conjugated secondary antibody (#A-11012; Invitrogen, Waltham, MA, USA) for 30 min at room temperature. Nuclei were counterstained using the VECTASHIELD Mounting Medium with DAPI (H-1200, Vector, Anaheim, CA, USA). The images were captured using a Keyence BZ-X800 fluorescence microscope.

### Ziehl–Neelsen staining

Samples of *M. avium*-infected HMDMs and formalin-fixed paraffin-embedded tissue sections were stained with carbol fuchsin (#41122, Muto Pure Chemicals, Osaka, Japan) for 30 min. After staining, the slides were rinsed with tap water, decolorized with 70% ethanol containing 1% HCl, and rinsed again with tap water. The slides were then counterstained with hematoxylin for 1 min, followed by rinsing, dehydration, and mounting.

### Multiplexed immunohistochemical consecutive staining on a single slide (MICSSS)

MICSSS was performed as previously described [14]. Briefly, serial rounds of immunohistochemical staining were performed on a single formalin-fixed, paraffin-embedded (FFPE) tissue section. After each round of staining, chromogenic signals were scanned using a NanoZoomer digital slide scanner (Hamamatsu Photonics, Shizuoka, Japan) and then removed using a mild antibody and chromogen stripping protocol. Staining was performed using different antibodies. This cycle was repeated to detect multiple markers in the same tissue section. The acquired images were aligned and analyzed using the HALO image analysis software program (Indica Labs, NM, USA) to generate pseudo-fluorescent composite images and assess marker distribution within the tissue microenvironment.

### HALO analysis

Quantification of marker-positive cells was performed using the Highplex FL module in the HALO image analysis software program. Multichannel immunofluorescence images were analyzed to detect and quantify cells expressing specific markers. Regions of interest (ROIs) were defined based on tissue classification using the HALO Tissue Classifier module, which applies a machine-learning algorithm to distinguish different tissue compartments based on histomorphological features and marker expression patterns. Individual cells were identified using nuclear segmentation and marker positivity was determined using user-defined fluorescence intensity thresholds. The number and spatial distribution of positive cells were automatically calculated and exported for the statistical analysis.

### *M. avium* intratracheal infection mouse model

The experimental model was established using 6- to 8-week-old male BALB/c mice. *M. avium* (2 × 10^5^ CFU (colony-forming units)/mouse) in 50 µL of sterile saline was administered intratracheally. Mice were euthanized 6 weeks after infection. After dissecting, the lungs were fixed in 10% neutral-buffered formalin and embedded in paraffin. In the PD-L1 antibody treatment experiment, mice were randomly assigned to the PD-L1 antibody treatment group or the isotype control group by drawing lots. Group allocation was conducted by an investigator who was not involved in the infection procedures, data collection, or statistical analysis. The sample size for each experimental group was determined based on previous studies using similar *M. avium* infection models. Mice in which NTM infection was not detected by Ziehl–Neelsen staining of lung tissues after dissection were excluded from the study. No blinding was performed during the procedures or outcome assessment because the differences in treatment groups were evident due to the experimental procedures.

### Statistical analysis

Prism software (GraphPad Software, San Diego, CA, USA) was used for statistical analyses. All data are representative of two or three independent experiments. Center values represent the mean, and error bars indicate the standard deviation (SD). Differences between groups were examined for statistical significance using the two-sided Mann–Whitney U test. No adjustment for multiple comparisons was applied. In the animal experiments, differences in variance between the PD-L1 antibody treatment group and the isotype control group, as well as between infected and non-infected regions, were assessed using Levene’s test to confirm that no extreme differences in variability were present. Statistical significance was set at *p < 0.05; **p < 0.01; ***p < 0.001.

## Results

### *M. avium* infection increases PD-L1 gene expression in macrophages

**Fig. 1:**
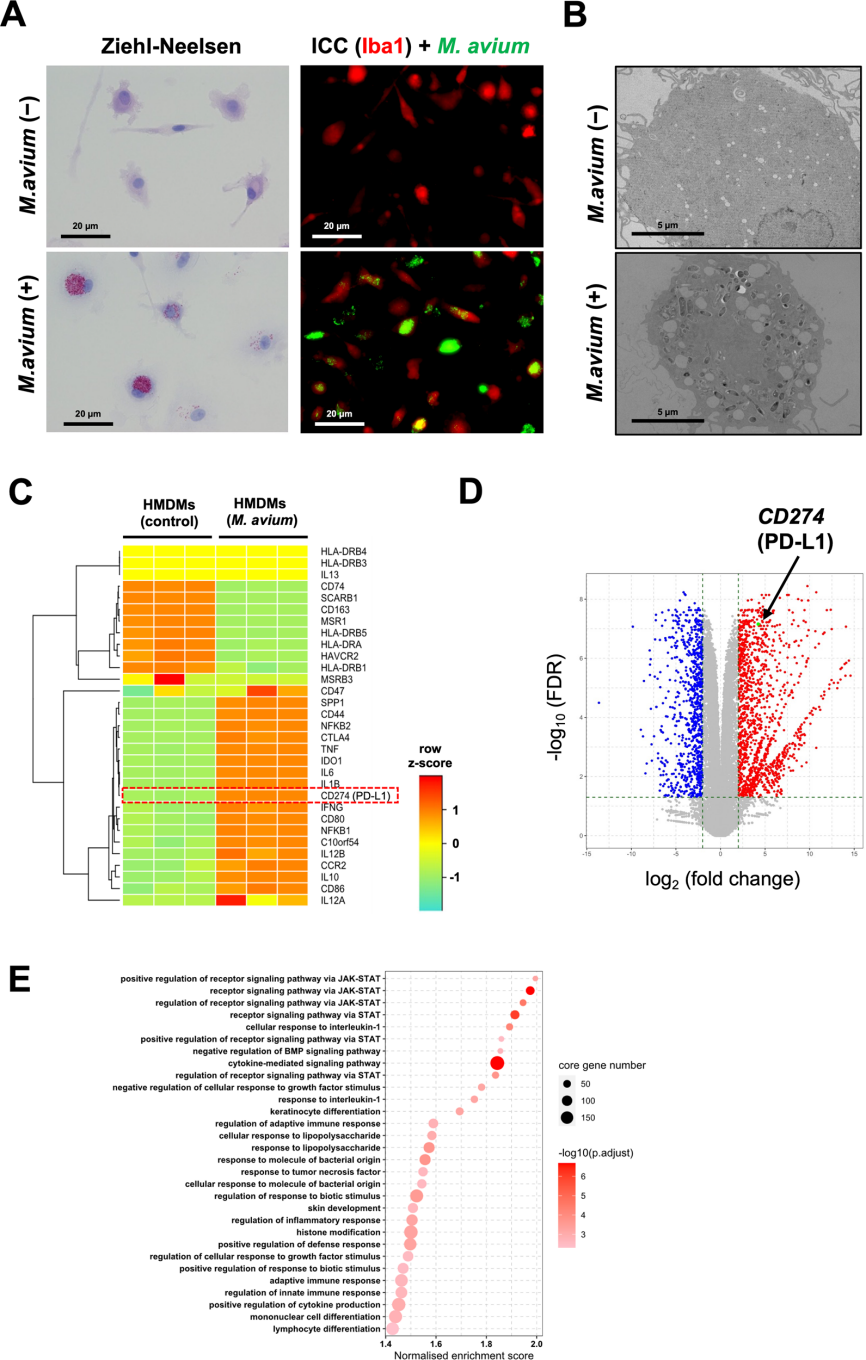
Effect of *Mycobacterium avium* infection on PD-L1 gene expression in human monocyte-derived macrophages (HMDMs). **A** Ziehl–Neelsen (Z–N) staining and immunocytochemistry image (red: Iba1, a pan-macrophages marker, green: GFP-expressing *M. avium*) in human monocyte-derived macrophages (HMDMs) after *M. avium* infection. Scale bar: 20 µm. **B** Electron microscopy images of HMDMs infected with *M. avium*. Scale bar: 5 µm. **C** Heatmap of selected gene expression in *M. avium*-infected HMDMs compared with non-infected controls. CPM values of the read count (adjusted by the library size of each sample with TMM normalization) of each gene were logarithmically transformed and scaled to *z*-scores. **D** Volcano plot for the results of the differentially expressed gene analysis between control and *M. avium*-infected HMDMs. The dot representing PD-L1 is highlighted. **E** Top 30 significant terms for the gene sets of gene ontology biological processes enriched in *M. avium-*infected HMDMs compared with non-infected controls with the lowest adjusted p values of the gene set enrichment analysis using the DEG analysis result. The dot representing each term is ordered according to the normalized enrichment score.

To examine the effects of NTM infection on gene expression in macrophages, we infected HMDMs with *M. avium*, a common infectious agent in Japan (Fig. 1A, B). *M. avium* infection enhanced PD-L1 gene expression in HMDMs (Fig. 1C, D), suggesting that *M. avium*-infected macrophages contribute to the formation of an immunosuppressive microenvironment via the PD-L1/PD-1 pathway. Furthermore, activation of the JAK-STAT signaling cascade was observed in HMDMs following *M. avium* infection (Fig. 1E). These findings, based on a bulk RNA-sequencing analysis, indicate that *M. avium* may induce PD-L1 expression in macrophages via the JAK-STAT1 signaling pathway, consistent with the well-established mechanism whereby IFN-γ-induced PD-L1 expression is regulated by STAT1 signaling in macrophages.

### *M. avium* infection increases PD-L1 protein expression in macrophages

**Fig. 2:**
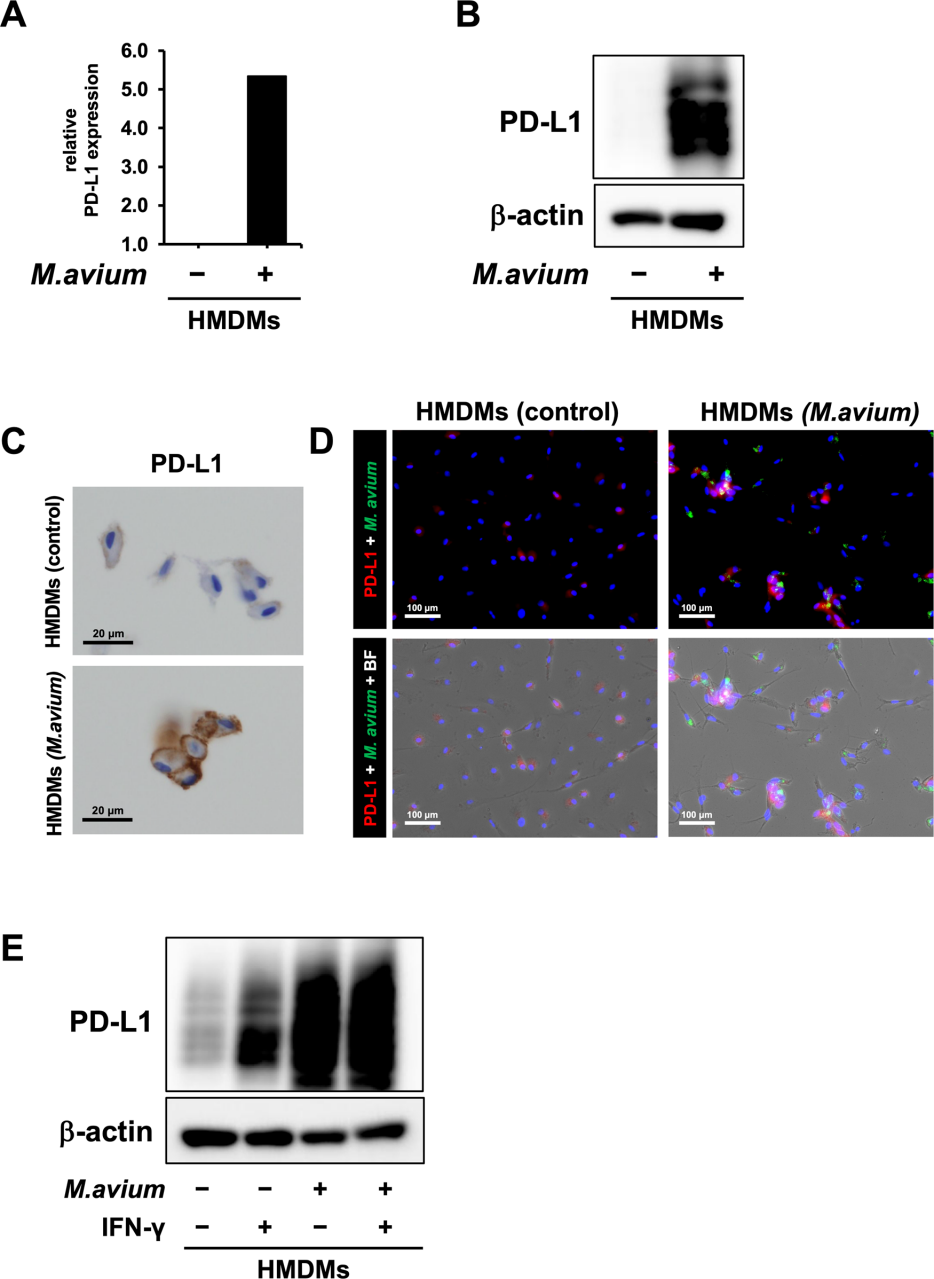
Effect of *Mycobacterium avium* infection on PD-L1 expression in human monocyte-derived macrophages (HMDMs). **A** PD-L1 mRNA expression in HMDMs infected with *M. avium* was measured using real-time quantitative PCR. **B** Protein expression of PD-L1 and β-actin in HMDMs infected with *M. avium* was measured using western blotting. **C** Immunostaining image of PD-L1 in HMDMs infected with *M. avium*. Scale bar: 20 µm. **D** Immunocytochemistry image　(red: PD-L1, green: GFP-expressing *M. avium*) in HMDMs. Scale bar: 100 µm. **E** Protein expression of PD-L1 and β-actin in *M. avium*-infected HMDMs with or without IFN-γ (10 ng/mL) was measured using western blotting.

Next, we investigated the effects of NTM infection on PD-L1 expression in macrophages. As shown in Fig. 2A. *M.* *avium* infection increased PD-L1 mRNA expression in HMDMs. The enhanced PD-L1 protein expression in *M. avium*-infected HMDMs was also confirmed by Western blotting (Fig. 2B), immunohistochemical staining (Fig. 2C), and immunofluorescence staining (Fig. 2D), indicating that *M. avium* infection induces PD-L1 expression in macrophages. Notably, PD-L1 induction by *M. avium* infection was comparable to, or in some instances exceeded, that observed after IFN-γ stimulation (Fig. 2E).

### *M. avium* infection activates the STAT1, MAPK and NF-κB signaling pathway in macrophages

**Fig. 3:**
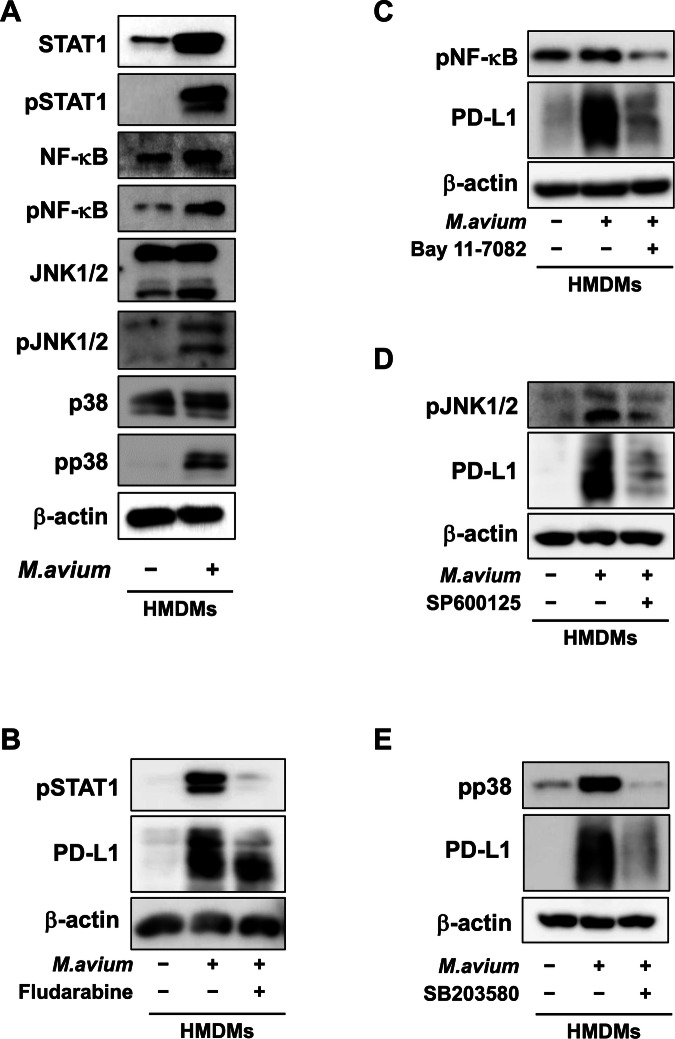
Effect of *Mycobacterium avium* infection on PD-L1 expression via STAT1, NF-κB and MAPK signaling pathways in human monocyte-derived macrophages (HMDMs). **A** Western blot analysis of phosphorylated and total forms of STAT1, NF-κB, JNK1/2, p38, along with β-actin, in HMDMs infected with *M. avium*. **B** Effect of fludarabine (500 µM; STAT1 inhibitor) on PD-L1, phosphorylated STAT1, and β-actin expression in *M. avium*-infected HMDMs. **C** The effect of Bay 11-7082 (10 µM; NF-κB inhibitor) on PD-L1, phosphorylated NF-κB, and β-actin expression in *M. avium*-infected HMDMs. **D** Effect of SP600125 (50 µM; JNK inhibitor) on PD-L1, phosphorylated JNK1/2, and β-actin expression in *M. avium*-infected HMDMs. **E** Effect of SB203580 (0.5 µM; p38 inhibitor) on PD-L1, phosphorylated p38, and β-actin expression in *M. avium*-infected HMDMs.

As IFN-γ induces PD-L1 expression by STAT1 activation, we next measured the effect of *M. avium* infection on STAT1 activation. As shown in Fig. 3A, *M. avium* infection activated STAT1, and STAT1 inhibitors suppressed *M. avium* infection-induced PD-L1 expression in HMDMs (Fig. 3B), indicating that STAT1 signaling pathway is associated with PD-L1 expression in macrophages following *M. avium* infection.

Since Toll-like receptor (TLR) signaling contributes to PD-L1 expression in monocytes/macrophages, we next measured the effect of *M. avium* infection on both MAPK activation and NF-κB activation in HMDMs. As shown in Fig. 3A, M*. avium* infection induced activation of MAPK (p38, JNK) and NF-κB in HMDMs. Additionally, NF-κB and MAPK (p38, JNK) inhibitors interfered with PD-L1 expression induced by *M. avium* infection (Fig. 3C–E), suggesting that both MAPK and NF-κB signaling pathways are associated with PD-L1 expression in macrophages following *M. avium* infection. These results indicate that NF-κB signaling pathway and STAT1 signaling cascade play an important role in the *M. avium* infection-induced PD-L1 expression in macrophages. All these findings were obtained through Western blotting, highlighting the activation and inhibition profiles of the related signaling pathways.

### *M. avium* infection enhances PD-L1 expression on macrophages in a mouse model

**Fig. 4:**
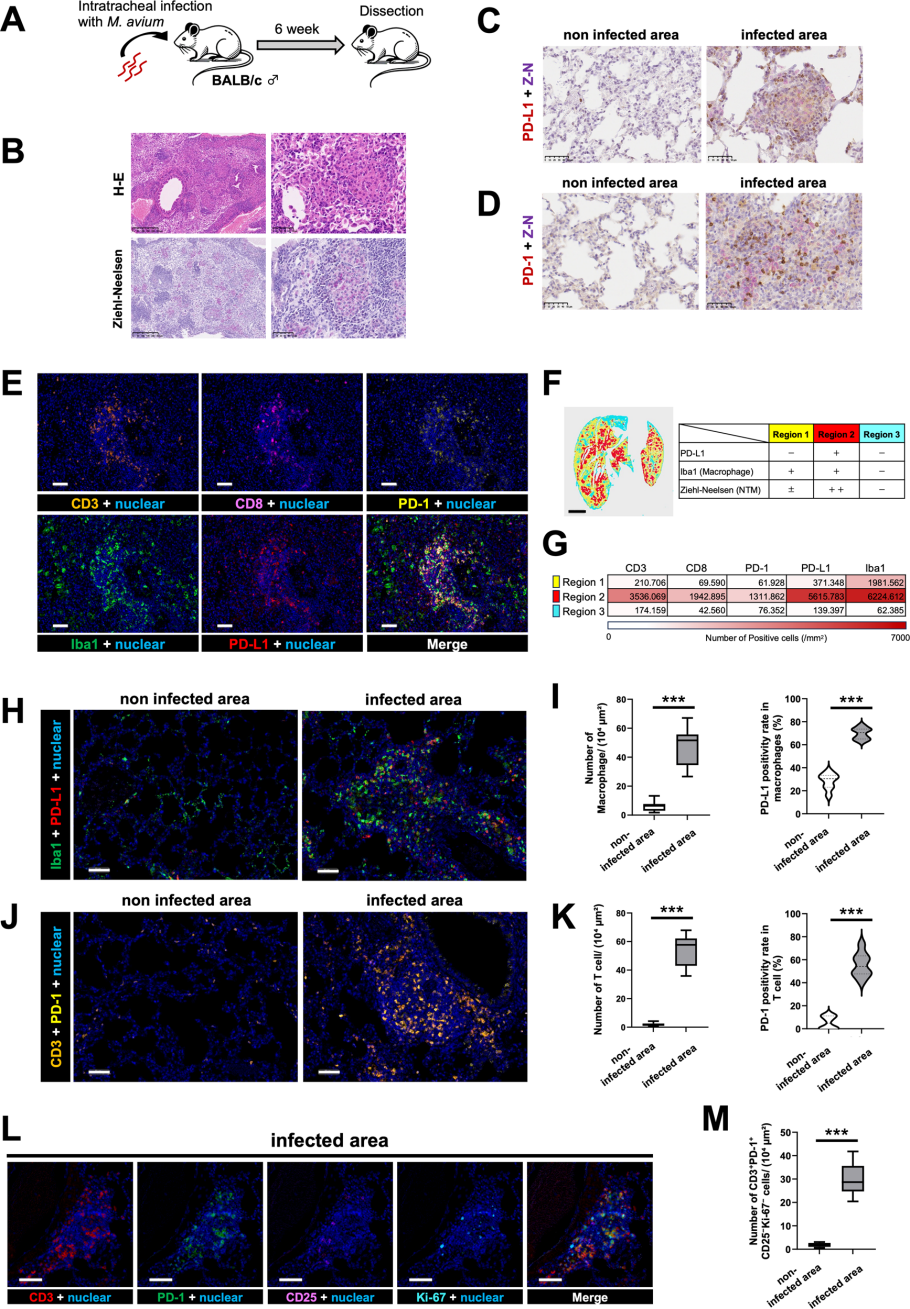
Evaluation of the tissue microenvironment in a mouse model of *Mycobacterium avium* infection. **A** Schematic diagram of the NTM-infected mouse model. Six-week-old male BALB/c mice were intratracheally infected with *M. avium* and euthanized 6 weeks later. **B** Histological image of lung tissue stained with hematoxylin and eosin (H&E) and Ziehl–Neelsen (Z–N). Scale bars: 250 µm and 50 µm. **C** Double staining image of PD-L1 immunostaining with Z–N. Scale bar: 50 µm. **D** Double staining image of PD-1 immunostaining with Z–N. Scale bar: 50 µm. **E** Representative images obtained by multiplexed immunohistochemical consecutive staining on single slide (MICSSS) with pseudo-fluorescence transformation, showing CD3, CD8, PD-1, Iba1, and PD-L1, along with merged images, in lung tissue from NTM-infected mice. Scale bar: 50 µm. **F** Positional plot of the neighborhoods color-coded by the region type. Scale bar: 2 mm. **G** Heatmap of the number of positive cells in each region normalized by the analyzed area. These results are representative of two independent experiments. **H** MICSSS pseudo-fluorescence image for Iba1 and PD-L1 (red: PD-L1; green: Iba1). Scale bar: 50 µm. **I** Comparison of the number of Iba1^+^ cells and PD-L1 positivity rate in macrophages between non-infected and infected areas using the HALO image analysis software program (*n* = 8 per group). **J** MICSSS pseudo-fluorescence image for CD3 and PD-1 (orange: CD3; yellow: PD-1). Scale bar: 50 µm. **K** Comparison of the number of CD3^+^ cells and the PD-1 positivity rate in lymphocytes between non-infected and infected areas using the HALO image analysis software program (*n* = 8 per group). **L** MICSSS pseudo-fluorescence images of CD3, PD-1, CD25, and Ki-67, along with merged images, in lung tissue from NTM-infected mice. Scale bar: 50 µm. **M** Comparison of the number of PD-1^+^, CD25^–^, Ki-67^–^ cells between non-infected and infected areas using the HALO image analysis software program (*n* = 8 per group). Data are expressed as mean ± SD; *p < 0.05; **p < 0.01; ***p < 0.001. Differences between groups were examined for statistical significance using the Mann-Whitney *U* test.

We investigated the effect of *M. avium* infection on PD-L1 expression in macrophages using a mouse model of infection (Fig. 4A). In this model, *M. avium* infection was detected in alveolar macrophages, and infected areas were identified using Ziehl–Neelsen staining (Fig. 4B). In regions with a high bacterial burden, strong expression of PD-L1 and PD-1 was observed, as confirmed by double staining with immunohistochemistry and Ziehl–Neelsen staining (Fig. 4C, D).

To further examine alterations in the tissue microenvironment associated with *M. avium* infection, we analyzed the spatial distribution of lymphocytes and macrophages as well as expression patterns of PD-L1 and PD-1. These analyses were performed using a combination of multiplexed immunohistochemical consecutive staining on single slide (MICSSS)[14] and pseudo-fluorescence imaging of brightfield images generated by the HALO software program. Based on the degree of NTM infection and macrophage distribution, lung tissues were classified into distinct regions (Fig. 4E, F). In Region 2, characterized by a high bacterial burden and abundant PD-L1-positive macrophages, a significant increase in T cell infiltration and PD-1-positive cells was observed (Fig. 4G). These findings suggest that NTM-infected macrophages may contribute to the induction of T cell exhaustion in infected microenvironments. To further investigate this possibility, we quantitatively assessed macrophage infiltration and PD-L1 expression, along with T cell infiltration and PD-1 expression, in both NTM-infected and non-infected tissue regions.

As a result, we observed a higher infiltration of macrophages and increased PD-L1 positivity in infected areas (Fig. 4H, I). Furthermore, there was greater infiltration of CD3-positive T cells with a higher rate of PD-1 positivity in infected areas (Fig. 4J, K). Notably, there was a marked increase in T cells exhibiting a highly exhausted phenotype (PD-1^+^, CD25^–^, Ki-67^–^) (Fig. 4L, M). These findings suggest that in regions with a high NTM infection burden, enhanced macrophage infiltration and PD-L1 expression correlate with increased PD-1 expression in T cells, indicating T cell exhaustion.

### Non-tuberculous mycobacteria (NTM) infection enhances PD-L1 expression on macrophages in human tissues

**Fig. 5:**
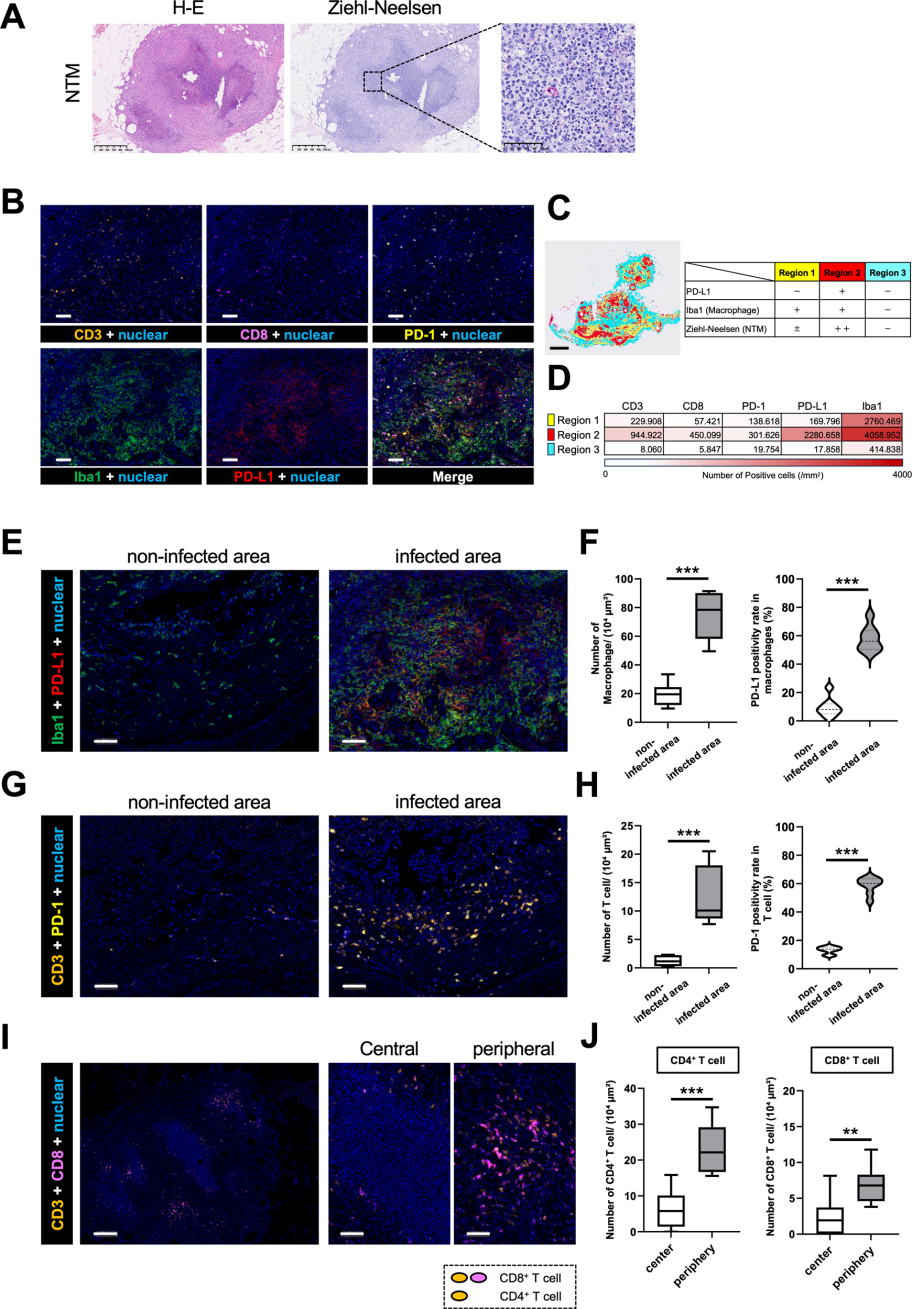
Evaluation of microenvironment in human tissues infected with non-tuberculous mycobacteria (NTM)*.* **A** Disseminated NTM-infected skin tissues were determined using hematoxylin and eosin (H&E) and Ziehl–Neelsen (Z–N) staining. Scale bars: 500 µm and 50 µm. **B** Representative images obtained by Multiplexed Immunohistochemical Consecutive Staining on Single Slide (MICSSS) with pseudo-fluorescence transformation, showing CD3, CD8, PD-1, Iba1, and PD-L1, along with merged images, in NTM-infected skin tissue. Scale bar: 50 µm. **C** Positional plot of the neighborhoods color-coded by the region type. Scale bar: 1 mm. **D** Heatmap of the number of positive cells in each region normalized by the analyzed area. These results are representative of two independent experiments. **E** MICSSS pseudo-fluorescence image for Iba1 and PD-L1 (red: PD-L1; green: Iba1). Scale bar: 50 µm. **F** Comparison of the number of Iba1^+^ cells and the PD-L1 positivity rate in macrophages between non-infected and infected areas using HALO image analysis software program (*n* = 8 per group). **G** MICSSS pseudo-fluorescence image for CD3 and PD-1 (orange: CD3; yellow: PD-1). Scale bar: 50 µm. **H** Comparison of the number of CD3^+^ cells and the PD-1 positivity rate in lymphocytes between non-infected and infected areas using HALO image analysis software program (*n* = 8 per group). **I** MICSSS pseudo-fluorescence images for CD3 and CD8 in central and peripheral regions of infected tissue (orange: CD3; pink: CD8). Scale bars: 200 µm and 50 µm. **J** Numbers of CD3^+^/CD8^−^ cells (CD4^+^ T cell) and CD3^+^/ CD8^+^ cells (CD8^+^ T cell) were analyzed in the central and peripheral regions of the infected site using HALO image analysis software program (*n* = 8 per group). Data are expressed as mean ± SD; *p < 0.05; **p < 0.01; ***p < 0.001. Differences between groups were examined for statistical significance using the Mann-Whitney *U* test.

We next examined PD-L1 expression in macrophages at sites of infection in cases of disseminated mycobacterial disease (Figs. 5A and S2A). Immunohistochemical analysis of lesions caused by both NTM and Mycobacterium tuberculosis (TB) revealed presence of PD-L1-positive macrophages and PD-1-expressing lymphocytes, indicating that immune checkpoint molecules are upregulated within the infected tissue microenvironment (Fig. S1A, B). To investigate alterations in the tissue microenvironment, we analyzed the spatial distribution of lymphocytes and macrophages, as well as expression patterns of PD-L1 and PD-1. These data were obtained using MICSSS and pseudo-fluorescence imaging based on brightfield images generated by HALO. Based on the extent of infection and macrophage distribution, tissues were classified into distinct regions (Figs. 5B, C and S2B, C). In Region 2, defined by a high bacterial burden and abundant PD-L1-positive macrophages, greater T cell infiltration and increased PD-1-positive cells were observed (Figs. 5D and S2D). These findings suggest that, in human tissue as well, NTM-infected macrophages may play a key role in promoting T cell exhaustion within infected niches. To validate this hypothesis, we quantitatively assessed macrophage infiltration and PD-L1 expression, as well as T cell infiltration and PD-1 expression, in both infected and non-infected regions.

Quantitative analysis revealed significantly higher macrophage infiltration and PD-L1 positivity in infected areas in comparison to non-infected areas (Figs. 5E, F and S2E, F). Similarly, T cell infiltration and PD-1 expression were both elevated in infected areas (Figs. 5G, H and S2G, H). Furthermore, analysis of T cell distribution between the center and the periphery of mycobacterial infection sites revealed significantly reduced infiltration of both CD4-positive and CD8-positive T cells in the center (Figs. 5I, J and S2I, J). These findings suggest that at mycobacterial infection sites, increased PD-L1 expression in macrophages, along with the resulting immune exhaustion of T cells, contributes to suppression of T cell infiltration into the central region of the infection site.

### PD-1/PD-L1 blockade promotes T cell reactivation in a mouse model

**Fig. 6:**
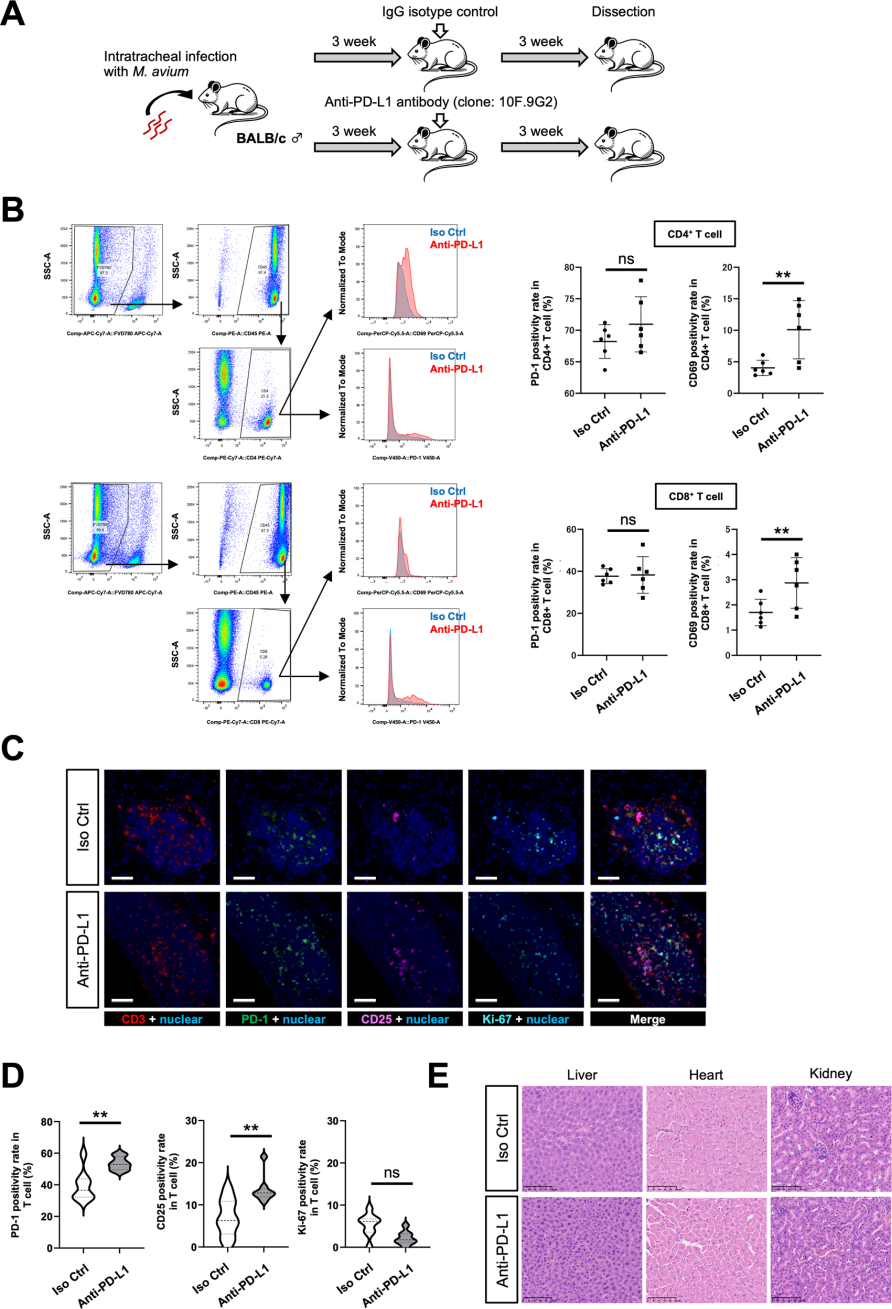
Evaluation of the T cell function following PD-1/PD-L1 blockade in a mouse model of *Mycobacterium avium* infection. **A** Schematic diagram of the NTM-infected mouse model. Six-week-old male BALB/c mice were intratracheally infected with *M. avium* to establish a respiratory infection model. At 3 weeks post-infection, mice received intraperitoneal injections of either anti-mouse PD-L1 monoclonal antibody (clone 10F.9G2) or rat IgG2 isotype control antibody (clone LTF-2) at 10 mg/kg. Three weeks after antibody administration, all animals were euthanized for downstream immunological analyses. **B** Flow cytometric analysis of PD-1 and CD69 expression on T cells in anti-PD-L1-treated and control groups. **C** Representative images obtained by multiplexed immunohistochemical consecutive staining on single slide (MICSSS) with pseudo-fluorescence transformation, showing CD3, PD-1, CD25, and Ki-67, along with merged images, in lung tissue from NTM-infected mice. Scale bar: 50 µm. **D** Quantification of PD-1, CD25, and Ki-67 positivity rates in T cells from anti-PD-L1-treated and untreated groups using the HALO image analysis software program (*n* = 8 per group). **E** Representative hematoxylin and eosin (H&E) staining of major organs after treatment with or without anti-PD-L1 antibody. Scale bar: 100 µm. Data are expressed as the mean ± SD; *p < 0.05; **p < 0.01; ***p < 0.001. Differences between groups were examined for statistical significance using the Mann-Whitney *U* test.

Finally, we evaluated the effect of blocking the PD-1/PD-L1 pathway on immune activation in a mouse model of NTM infection (Fig. 6A). Administration of the anti-PD-L1 antibody enhanced expression of CD69, an activation marker, on CD4^+^ and CD8^+^ T cells in NTM-infected lung tissues (Fig. 6B). In addition, the proportion of PD-1- and CD25-positive T cells was elevated in lung tissues following treatment (Fig. 6C, D), without immune-related adverse events (irAEs) (Fig. 6E). These findings suggest that PD-1/PD-L1 blockade is a potential therapeutic strategy for MAC infection by T cell reactivation.

## Discussion

Currently, most patients with TB, except for a few patients with multidrug-resistant TB, are expected to be curable. However, no treatment for NTM has yet been established. Although the pathogenicity of NTMs is weaker than that of *Mycobacterium tuberculosis*, antibiotic treatment lasts for 1–2 years and may continue for an extended period. Therefore, new therapeutic strategies are required.

In this context, there is growing interest in host-directed therapy, which aims to enhance the immune ability of the host against microbes or to improve the effectiveness of antibiotic treatment [15, 16]. Immune checkpoint inhibition therapy is gaining attention in cancer treatment and is a novel approach that enhances antitumor immunity by disrupting immune suppression mechanisms. This is achieved by administering antibodies that bind to immunosuppressive receptors or ligands, thereby activating T cell responses. Immune checkpoint inhibitors (ICIs) have been reported to activate immune responses against pathogenic microorganisms. For example, PD-1-blocking antibodies enhance survival in mice infected with *Histoplasma capsulatum* [17], and PD-1-deficient mice show improved survival follwing infection with *Mycobacterium bovis* bacillus Calmette-Guérin [18]. Furthermore, partially blocking PD-1 and PD-L1 with antagonizing antibodies in the stimulation assay significantly increased IFN-γ production and decreased apoptosis of T cells in patients with MAC-induced lung disease, suggesting that ICIs might improve lymphocytic secretion of IFN-γ and reduce apoptosis in these patients [10]. Administration of nivolumab, a well-known ICI, improves outcomes in patients with pulmonary NTM disease by regulating T cell activation [13]. However, as there are reports of tuberculosis or opportunistic infections following ICI administration, more detailed studies elaborating on the effects of ICIs on microorganisms are needed [19–22].

Macrophages polarize in response to various stimuli, including bacterial infections^23^. Specifically, infections with facultative intracellular pathogens, such as *Salmonella*, *Listeria*, and TB, lead to polarization in a population of classically activated macrophages, commonly called M1-like macrophages [24]. Alternatively, activated macrophages, also known as M2-like macrophages, are induced by Th2 cytokines, such as IL-4, IL-10, and IL-13 [25, 26]. They play important roles in immunosuppression, tissue remodeling, and angiogenesis [27]. Tumor-associated macrophages (TAMs) often exhibit M2-like characteristics and promote tumor progression [28]. TAMs express high levels of PD-L1 and are involved in immunosuppression [29]. According to RNA sequencing results, macrophages infected with *M. avium* showed increased expression of genes related to inflammatory cytokines, indicating an M1-like phenotype. However, *M. avium*-infected macrophages exhibited increased PD-L1 expression, demonstrating a phenotype with pro-inflammatory and anti-inflammatory properties (Fig. 1). Detailed macrophage phenotype analysis is necessary to elucidate the pathogenesis of NTM infections.

The NF-κB pathway is known to contribute to PD-L1 expression in several malignant tumors, including non-small cell lung cancer and cervical cancer, thereby promoting tumor progression [30, 31]. Activation of the NF-κB pathway is involved in upregulation of PD-L1 expression in immunosuppressive macrophages, forming the fundamental requirement for primary tumor metastasis [32]. In the current study, we revealed that *M. avium*-infected macrophages enhanced PD-L1 expression via NF-κB and MAPK activation, suggesting that these macrophages may escape the immune response of T cells.

In the NTM-infected mouse model, PD-L1 blockade enhanced expression of T cell activation markers, including CD69 and CD25 (Fig. 6B–D). Additionally, there was a discernible trend toward enhanced PD-1 expression (Fig. 6B–D), suggesting heightened T cell activation and potential regulatory feedback following immune checkpoint inhibition.

It is well established that engagement of PD-1 by its ligand PD-L1 triggers intracellular inhibitory signaling cascades that attenuate T cell proliferation, cytokine production, and cytotoxic function, thereby serving as a critical mechanism to limit excessive immune activation and prevent autoimmunity [33]. However, in the context of chronic infections and malignancies, persistent activation of the PD-1/PD-L1 axis contributes to profound immune suppression, facilitating immune evasion by pathogens and tumor cells. Notably, emerging evidence indicates that PD-1 expression is also upregulated in functionally reactivated T cells following immune checkpoint blockade therapy, suggesting dynamic and context-dependent regulation of this inhibitory pathway [34–36]. Therefore, the reactivation of T cells observed following PD-L1 blockade in NTM-infected mice, as demonstrated in this study, was further substantiated by the upregulated expression of T cell activation markers and PD-1.

Therefore, future investigations should focus on developing combinatorial strategies that simultaneously target multiple immune checkpoints, enhance macrophage-mediated bactericidal activity via the adjunctive use of conventional antimicrobials, and incorporate additional immunomodulatory approaches, such as blockade of immunosuppressive cytokines or modulation of host metabolic pathways, to achieve a more robust and comprehensive therapeutic effect.

In summary, *Mycobacterium avium*-infected macrophages exhibit an M1-like proinflammatory phenotype while concurrently upregulating PD-L1, a mechanism that likely contributes to immune evasion by modulating T cell responses. The induction of PD-L1 is associated with activation of key inflammatory signaling pathways, including NF-κB and MAPK, suggesting that pharmacological targeting of these pathways may enhance host antimicrobial immunity. Future studies should explore rational combination strategies involving additional immune checkpoint inhibitors and conventional antimicrobials to improve therapeutic efficacy against NTM infections.

## Supplementary information

Supplementary Figure 1

Supplementary Figure 2

Supplementary Figure Legends

Original Data

## Data Availability

The bulk RNA-seq data generated in this study are available from the corresponding author upon reasonable request.
