# Supplementary figures and images for: Mycobacterium avium infection induced PD-L1 overexpression in macrophages: a potential involvement with immune escape
Source: Cell Death Dis. 2026 Jan 9;17(1):15. doi: 10.1038/s41419-025-08165-z (PMC12789633; doi:10.1038/s41419-025-08165-z)

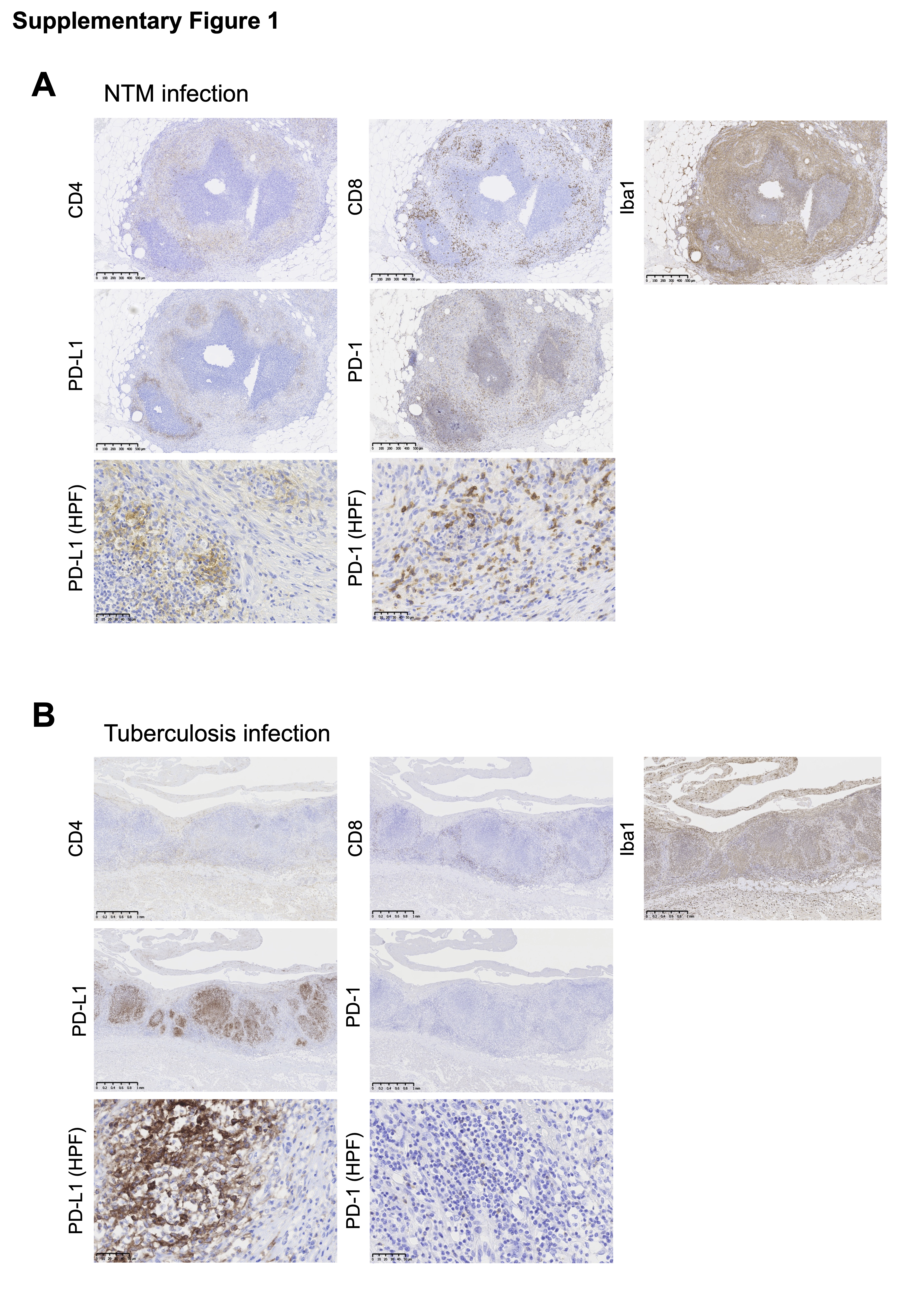

Supplement: Supplementary file 1 — Supplementary Figure 1 [file 41419_2025_8165_MOESM1_ESM.png]

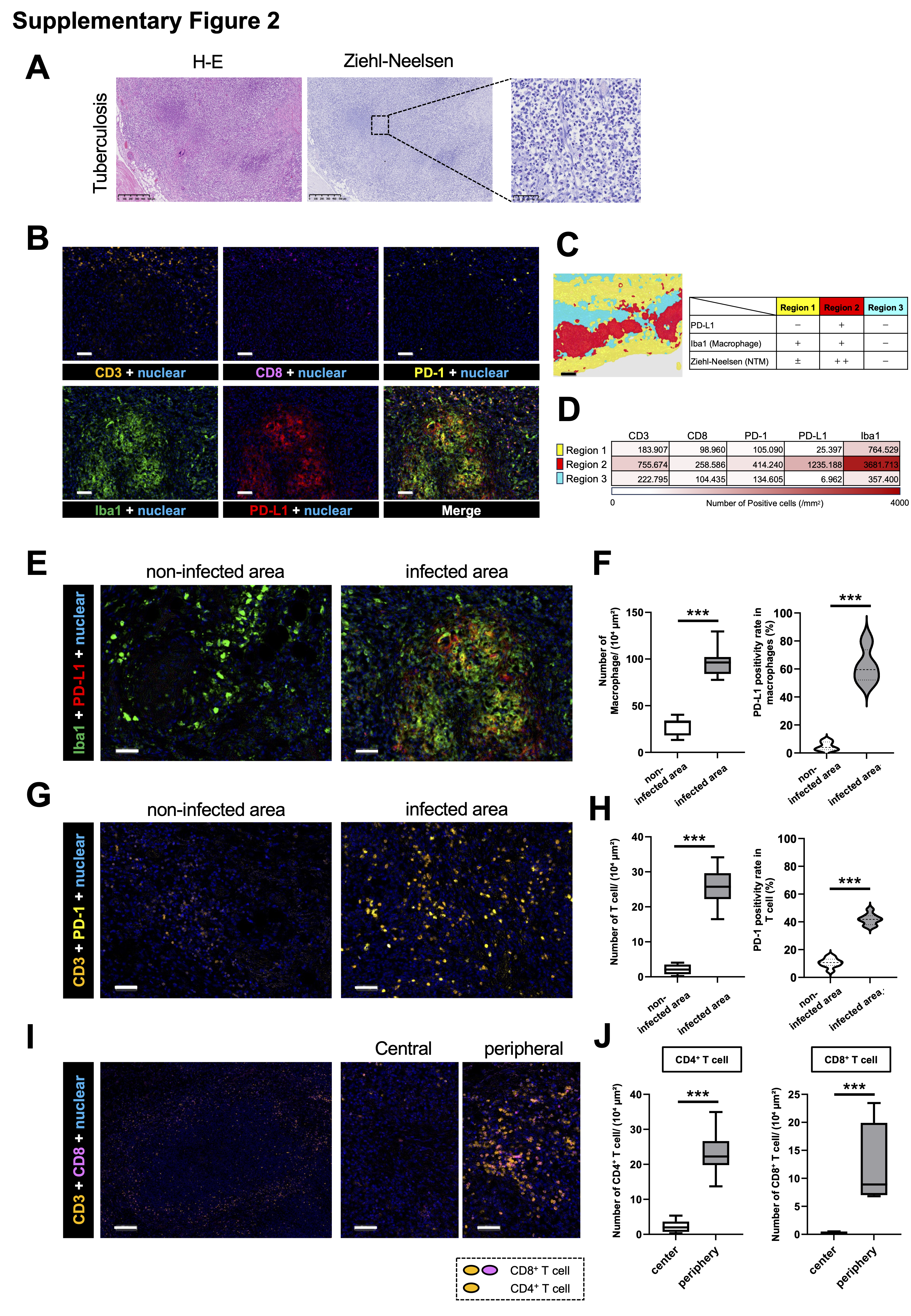

Supplement: Supplementary file 2 — Supplementary Figure 2 [file 41419_2025_8165_MOESM2_ESM.png]

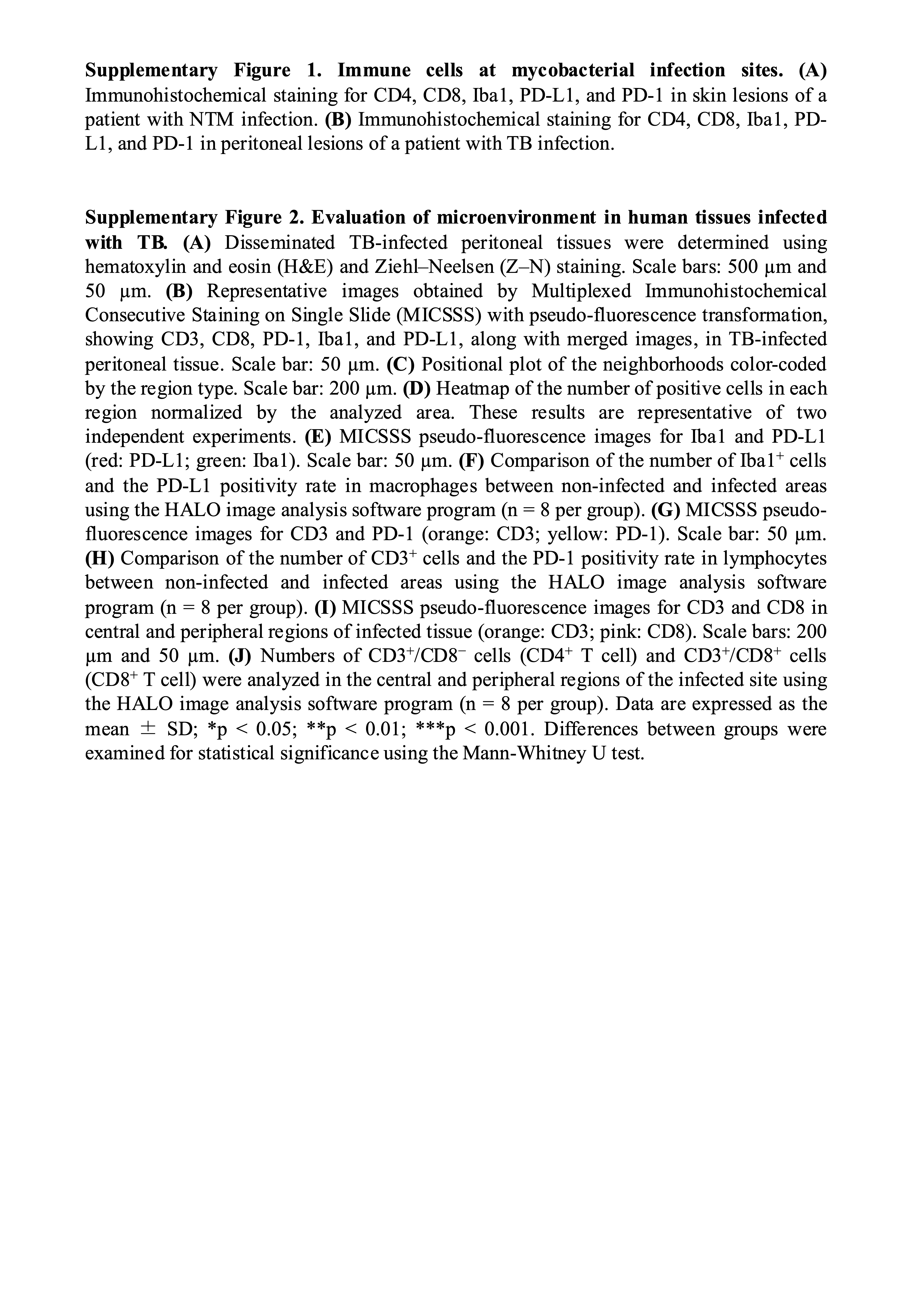

Supplement: Supplementary file 3 — Supplementary Figure Legends [file 41419_2025_8165_MOESM3_ESM.png]
